# Role of metal oxide nanoparticles in histopathological changes observed in the lung of welders

**DOI:** 10.1186/1743-8977-11-23

**Published:** 2014-05-13

**Authors:** Pascal Andujar, Angélique Simon-Deckers, Françoise Galateau-Sallé, Barbara Fayard, Gregory Beaune, Bénédicte Clin, Marie-Annick Billon-Galland, Olivier Durupthy, Jean-Claude Pairon, Jean Doucet, Jorge Boczkowski, Sophie Lanone

**Affiliations:** 1 Centre Hospitalier Intercommunal de Créteil, Service de Pneumologie et de Pathologie Professionnelle, 94000 Créteil, France; 2 INSERM, U955, Equipe 4, 94000 Créteil, France; 3 Université Paris Est-Créteil, Faculté de Médecine, 94000 Créteil, France; 4 Laboratoire de Physique des Solides, CNRS UMR 8502, 91400 Orsay, France; 5 CHU Caen, Service d’Anatomo-Pathologie, 14000 Caen, France; 6 INSERM, U1086, Faculté de Médecine, 14000 Caen, France; 7 Sorbonne Universités, UPMC Univ. Paris 06, UMR 7574, Laboratoire de Chimie de la Matière Condensée de Paris, F-75005 Paris, France; 8 CNRS, UMR 7574, Laboratoire de Chimie de la Matière Condensée de Paris, F-75005 Paris, France; 9 Collège de France, UMR 7574, Laboratoire de Chimie de la Matière Condensée de Paris, F-75005 Paris, France; 10 CHU Caen, Service de Santé au Travail et Pathologie Professionnelle, 14000 Caen, France; 11 Laboratoire d’Etude des Particules Inhalées, 75013 Paris, France; 12 CHU Henri Mondor, Service d’explorations fonctionnelles respiratoires, 94000 Créteil, France

**Keywords:** Welding, Occupational exposure, Metal oxide nanoparticle, Inflammation, Lung

## Abstract

**Background:**

Although major concerns exist regarding the potential consequences of human exposure to nanoparticles (NP), no human toxicological data is currently available. To address this issue, we took welders, who present various adverse respiratory outcomes, as a model population of occupational exposure to NP.

The aim of this study was to evaluate if welding fume-issued NP could be responsible, at least partially, in the lung alterations observed in welders.

**Methods:**

A combination of imaging and material science techniques including ((scanning) transmission electron microscopy ((S)TEM), energy dispersive X-ray (EDX), and X-ray microfluorescence (μXRF)), was used to characterize NP content in lung tissue from 21 welders and 21 matched control patients. Representative NP were synthesized, and their effects on macrophage inflammatory secretome and migration were evaluated, together with the effect of this macrophage inflammatory secretome on human lung primary fibroblasts differentiation.

**Results:**

Welding-related NP (Fe, Mn, Cr oxides essentially) were identified in lung tissue sections from welders, in macrophages present in the alveolar lumen and in fibrous regions. In vitro macrophage exposure to representative NP (Fe_2_O_3_, Fe_3_O_4_, MnFe_2_O_4_ and CrOOH) induced the production of a pro-inflammatory secretome (increased production of CXCL-8, IL-1ß, TNF-α, CCL-2, −3, −4, and to a lesser extent IL-6, CCL-7 and −22), and all but Fe_3_O_4_ NP induce an increased migration of macrophages (Boyden chamber). There was no effect of NP-exposed macrophage secretome on human primary lung fibroblasts differentiation.

**Conclusions:**

Altogether, the data reported here strongly suggest that welding-related NP could be responsible, at least in part, for the pulmonary inflammation observed in welders. These results provide therefore the first evidence of a link between human exposure to NP and long-term pulmonary effects.

## Background

Nanotechnologies represent a major stake of the 21^rst^ century. They are aimed at conceiving, characterizing and producing materials at the scale of the billionth meter; the nanometer. These nanomaterials (among which are particles with a diameter lesser than 100 nm - nanoparticles, NP) present new properties that make them particularly interesting to industrialists. The list of actual uses and applications for nanomaterials is already substantial, and will certainly become exponential in the next future. Such developments, in spite of potential benefits in numerous domains, i.e. in the field of nanomedicine, are accompanied by concerns regarding the potential consequences of human exposure (in the general population or in an occupational context), with particular threat at the respiratory level [1-3]. The need for toxicity evaluation is motivated by numerous studies already available in the literature demonstrating that animal exposure to engineered NP is followed, among other pathological manifestations, by the induction of a pulmonary inflammation, associated with lung remodelling [4-8]. However, so far, there is almost no study assessing the consequences of human exposure to manufactured NP. This point is critical since human exposure studies constitute the cornerstone of risk evaluation for health after (chronic) exposure to environmental agents, especially in the occupational context.

During their everyday occupation, welders are exposed to a complex aerosol of gases (e.g. carbon monoxide, ozone), and hazardous metal fumes composed of chain-like agglomerates of particles, with a primary size in the nanometer size range [9]. Indeed, up to 11% of the total mass, and 80% of the total number of particles emitted in welding fumes are NP [10,11]. Various adverse respiratory outcomes have been described in welders, among which inflammation and lung remodeling are largely described [12-16]. However, the underlying responsible mechanisms remain to be elucidated.

We therefore made the hypothesis that these abnormalities could result, at least partly, from an inflammatory effect directly induced by welding fume-issued NP present in the pulmonary tissue. Such hypothesis has never been studied in the literature so far, probably due to the technical challenges related to the analysis and characterization of NP tissular content in biological samples *in situ*. We therefore setup an original interdisciplinary study, combining the expertise of material science physicists, physico-chemists, biologists and medical doctors. A broad range of imaging and material science techniques (scanning/transmission electron microscopy (S/TEM), energy dispersive X-ray (EDX), and X-ray microfluorescence (μXRF)), was used to characterize the association between lung histological modifications and the presence of NP in 21 well-documented arc welders and matched unexposed individuals. Since this analysis revealed the presence of NP in macrophages in the alveolar lumen and in areas of fibrous tissue in welders, our physico-chemist partner chemically synthesized NP representative of those identified in pulmonary tissue (Fe_2_O_3_, Fe_3_O_4_, MnFe_2_O_4_ and CrOOH), to investigate their effects (in combination or not with exposure to cigarette smoke, as the majority of patients were smokers) on human macrophages. These exposures were performed in combination or not with exposure to cigarette smoke, as the majority of patients were smokers. Our results show that exposure to welding-related NP induce the production of a pro-inflammatory secretrome (increased production of CXCL-8, IL-1ß, TNF-α, CCL-2, −3, −4, and to a lesser extent IL-6, CCL-7 and −22). Moreover, all but Fe_3_O_4_ NP induce an increased migration of macrophages.Finally, there was no effect of macrophage pro-inflammatory secretome on human primary lung fibroblasts differentiation. These results provide therefore the first evidence of a link between human exposure to NP and long-term pulmonary effects.

## Results

### Clinical and histological characterization of patients

The clinical characteristics of the 42 patients included in our study are given in Table 1. Consistent with the population studied, female were significantly less represented in the welder group as compared to controls (p = 0.038). Otherwise, consistent with the selective matching of controls with welders, the two populations were similar for age and tobacco history.

**Table 1:** Clinicopathological characteristics of control and welder patients.

	**Controls**	**Welders**	**P value**
	**(n = 21)**	**(n = 21)**	
Mean age (years ± SD)	58.6 ± 10.8	58.5 ± 10.7	ns
**Gender**			
Female	6 (28.6%)	1 (4.8%)	0.038
Male	15 (71.4%)	20 (95.2%)	ns
**Tobacco smoking**			
Smoking status			ns
Current smokers	9 (43.0%)	6 (28.6%)	
Former smokers	9 (43.0%)	12 (57.1%)	
Never smokers	3 (14.0%)	3 (14.3%)	
Cumulative consumption (P-Y ± SD)	24.6 ± 29.0	22.2 ± 15.2	ns
Age at onset (years ± SD)	16.8 ± 3.4	18.3 ± 4.2	ns
Age at cessation (years ± SD)	51.6 ± 14.0	48.8 ± 11.2	ns
Duration (years ± SD)	32.6 ± 16.7	33.7 ± 10.3	ns
Duration since cessation (years ± SD)	5.7 ± 9.3	8.2 ± 10.4	ns
**Welding fumes exposure**			-
Positive occupational questionnaire	0	21	
Age at onset (years ± SD)	-	19.9 ± 7.6	
Age at cessation (years ± SD)	-	48.2 ± 9.9	
Duration (years ± SD)	-	27.0 ± 9.2	
Duration since cessation (years ± SD)	-	9.2 ± 10.4	

ns: not significant; Max: maximum; Min: minimum; P-Y: packs-years; SD: standard deviation.

**Figure 1:**
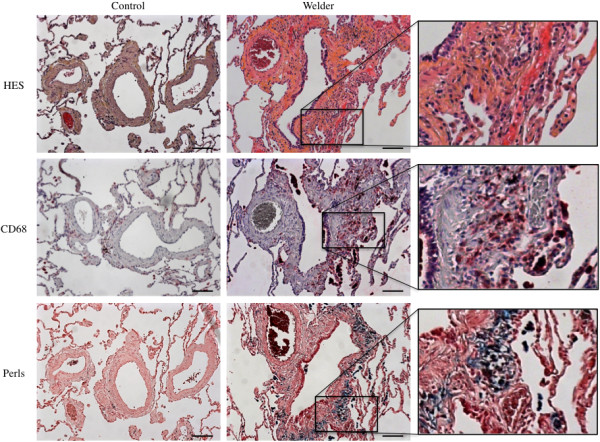
**Histological observation of lung tissue sections.** Representative optical microscopy images of lung tissue sections from a Control and a Welder. Serial sections were stained with hematoxylin-eosin-saffron (HES), CD68 or Perls. Scale bar: 100 μm. Inset: higher magnification of the welder sample.

CD68 staining revealed the presence of a higher number of macrophages in lung tissue sections from welders as compared to control patients, localized in the alveolar lumen as well as in the fibrous tissue (Figure 1). Importantly, black spots corresponding to aggregates of particles were essentially colocalized with CD68 staining suggesting a macrophagic internalization of these aggregates. The iron load, as revealed by Perls staining (Figure 1) and the quantification of siderophages (iron-laden macrophages) and ferruginous bodies (formed by macrophages) (Table 2), were significantly higher in welders as compared to control patients. Finally, lung fibrotic lesions were identified in both control and welders, but were significantly more severe in welders as compared to control patients irrespective of the histological localization (Figure 1 and Table 2). There was no difference for other respiratory lesions such as respiratory bronchiolectasis, bronchiolar dysplasia or metaplasia, or bronchiolitis between the two groups (Table 2).

**Table 2:** Histological analysis of lung tissue in control and welder patients

	**Controls**	**Welders**	**P value**
	**(n = 21)**	**(n = 21)**	
**Siderophages**			
Presence (n patients)	10	17	0.024
Number (Mean semi-quantitative score ± SD)^b^	0.71 ± 0.90	1.57 ± 1.17	0.019
**Ferruginous bodies (n patients)**			
Presence (n patients)	6	16	0.002
Number (Mean semi-quantitative score ± SD)^b^	0.43 ± 0.81	1.81 ± 1.57	0.002
**Fibrotic lesions (Number of patients)**			
Peribronchiolar	16	20	ns
Perivascular	14	20	0.018
Paraseptal	8	10	ns
Alveolar	3	5	ns
Diffuse interstitial	6	5	ns
Pleural	4	2	ns
**Severity of fibrosis**^**a**^			
(Mean semi-quantitative score ± SD)			
Global	2.29 ± 1.59	3.91 ± 1.51	0.003
Peribronchiolar	1.10 ± 0.77	1.86 ± 0.85	0.005
Perivascular	1.05 ± 0.92	1.81 ± 0.81	0.010
Alveolar and peribronchiolar	1.57 ± 1.12	2.67 ± 1.16	0.004
**Other respiratory lesions**			
Bronchiolectasis	16	13	ns
Bronchiolar metaplasia	2	0	ns
Bronchiolar dysplasia	1	5	ns
Respiratory Bronchiolitis – Intertitial Lung Disease	10	10	ns
Desquamative intertitial pneumonitis	12	9	ns
Endogenous Lipoid Pneumonitis	1	3	ns

ns: not significant; SD: standard deviation.

^a^Semi-quantitative score [4 classes = 0: absent; 1: rare; 2: frequent; 3: important]. Adapted from Roggli classification [54].

^b^Semi-quantitative score [5 classes = 0: 0/slide; 1: < 5/slide; 2: 5 to 10/slide; 3: 10 to 50/slide ; 4: > 50/slide].

### Patients’ particle burden characterization

#### Elemental mapping in lung tissue sections

**Figure 2:**
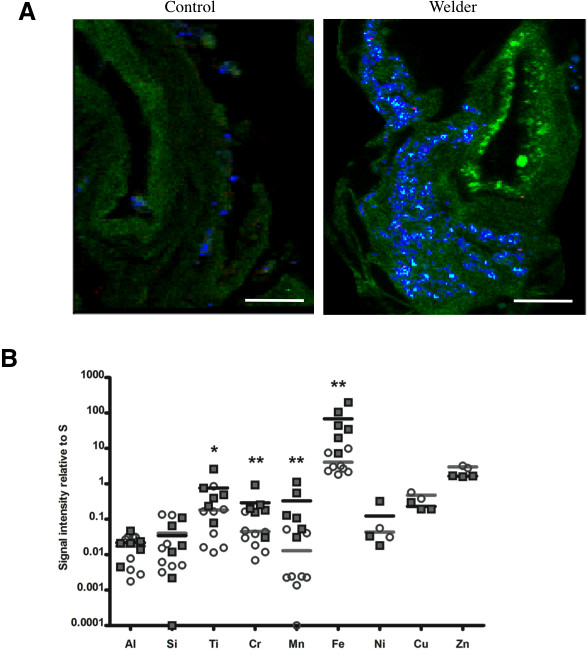
**Elemental mapping of lung tissue sections. A:** Representative elemental map obtained by X-ray micro-fluorescence (μXRF) in a Control and a Welder. Signal of sulphur: green. Signal of iron: blue. Signal of manganese: red. Scale bar: 100 μm. For each element, fluorescence intensities are proportional to the total amount of element. **B:** Quantification of the signal obtained for each element, given as a ratio to that of sulphur. *: p < 0.05 vs Control, **: p <0.01 vs Control, n = 6-8 per group. Open circles: Controls (grey horizontal bar: mean value). Black squares: Welders (black horizontal bar: mean value).

We first wanted to get a general overview of the elemental mapping at the global level of the tissue. To that matter, we used synchrotron-based X-ray microfluorescence (μXRF) and determined the spatial distribution of elements performing 100 × 100 μm maps. Figure 2A shows typical elemental μXRF maps obtained for sulfur (S), iron (Fe), and manganese (Mn) in control patients and welders. Since S represents a characteristic element of biological materials, the shape of the tissue can be drawn by the S map [17]. Using this approach, we observed a significant overload of Fe, Mn, chromium (Cr) essentially and to a lesser extent for titanium (Ti) in welders as compared to control patients (Figure 2B). This signal was essentially localized in the fibrous tissue (Figure 2), and to a lesser extent in the alveolar lumen (data not shown). Remarkably, we could observe that iron-rich zones were often co-localized with other element-rich zones, particularly Mn and Cr (Figure 2 and data not shown).

#### NP identification in lung tissue homogenates

**Figure 3:**
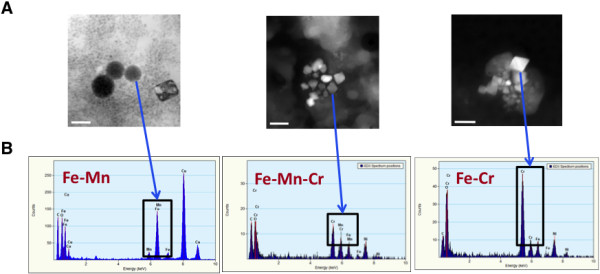
**Identification of NP in lung tissue homogenates. A:** Typical STEM images obtained in sodium hypochlorite-digested lung tissue samples from welders. Scale bar: 50 nm. **B:** EDX spectra of individual NP detected by STEM.

To determine individual exposure to asbestos bodies and non fibrous mineral particles, a mineralogical analysis of lung tissue homogenates was performed. These experiments confirmed the metallic overload in welder’s lung and identified a significantly higher mass concentration of total mineral and especially metallic particles in welders as compared to control patients (Table 3). Consistent with the initial selective matching of controls with welders, the two populations were similar for asbestos exposure characteristics. Finally, in order to characterize the size and chemical nature of the particles present in the pulmonary samples, we used a combined STEM-EDX (Scanning Transmission Electron Microscopy (STEM)-Energy Dispersive X-Ray (EDX)) analysis of lung tissue homogenates, and demonstrated that the particles are of nanometric size (20–25 nm individual diameter - STEM analysis Figure 3A), and confirmed the chemical nature (Fe, Mn, Cr essentially) and the co-localization of the chemical signals (EDX analysis, Figure 3B).

**Table 3:** Mineralogical analysis and asbestos exposure quantification of lung tissue homogenates in control and welder patients

	**Controls**	**Welders**	**P value**
	**(n = 21)**	**(n = 21)**	
**Dry lung tissue weight (mg)** (Mean ± SD)	24.8 ± 7.6	21.4 ± 8.0	ns
**Mineral particle concentration in lung tissue** (number of particles x 10^7^ per g; Mean ± SD)			
Total	70.2 ± 84.3	155.9 ± 148.8	0.012
Metallic	17.3 ± 22.7	73.9 ± 66.0	0.0005
Non metallic	48.8 ± 61.1	80.9 ± 105.1	ns
**Asbestos exposure**			
Positive occupational questionnaire	8	8	
AB/g dry lung tissue (median)	23,667	4,001	ns
[Min–Max]	[165–437,175]	[685–44,370]	ns
> 1000 AB/g dry lung tissue (n)	7	8	ns

AB: asbestos bodies; SD: standard deviation.

#### NP identification in lung tissue sections

**Figure 4:**
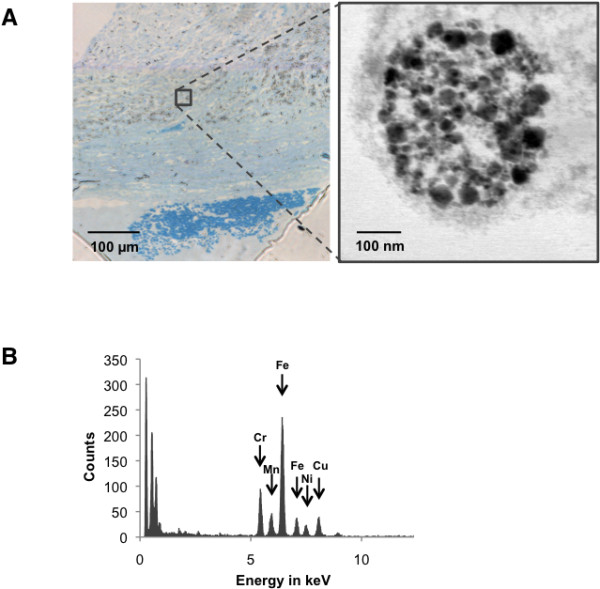
**Identification of NP in lung tissue sections. A:** Representative optical microscopy image of a thin section (Azur II staining) of a welder. Inset: higher magnification of a macrophage-containing fibrous region, observed by TEM. **B:** Representative EDX spectrum of individual NP detected by TEM.

Following the detection of NP in the lung tissue homogenates, we questioned the identification of these NP *in situ* at the nanometer scale and characterized their localization in lung tissue sections from patients, especially in areas of lung remodeling. We therefore proceeded to a combined TEM-EDX analysis of lung tissue sections and observed that, in comparison with control patients, large quantities of NP were localized in macrophages residues, and was present in the fibrous tissue of welders (Figure 4). In accordance with the results obtained in lung tissue homogenates, these NP presented a spherical shape, and a diameter centered around 20–25 nm. The chemical nature of the complex metal oxide NP in the welders lungs were mainly Fe, Cr and/or Mn (and aluminium (Al), silicium (Si), and nickel (Ni) to a lesser extent), whereas it was essentially Al and Si NP in the control patients (data not shown).

Overall, these different material science techniques allow us to demonstrate the presence of Fe, Cr and/or Mn NP *in situ* in lung tissue from welders, especially in macrophages.

### Synthesis of representative NP

**Figure 5:**
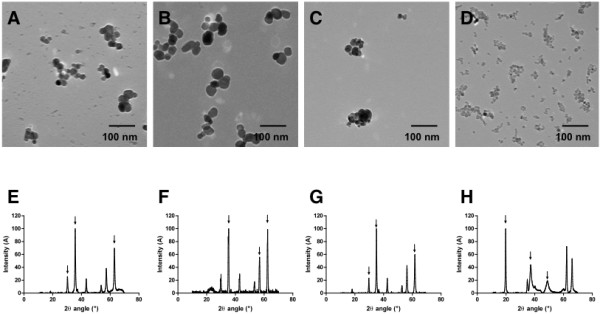
**Characterization of welding-representative NP. A-D:** Representative TEM images of Fe_2_O_3_**(A)**, Fe_3_O_4_**(B)**, MnFe_2_O_4_**(C)** and CrOOH **(D)** NP. **E-H:** Corresponding XRD spectra obtained for each NP. The three important 2θ values (arrow) of XRD spectra were use to identified synthesized NP as Fe_2_O_3_**(E)**, Fe_3_O_4_**(F)**, MnFe_2_O_4_**(G)** and CrOOH **(H)**.

In order to answer the question whether the metal NP identified above have a causal role in the development of pulmonary alterations observed in welders, we next chemically synthesized 4 NP representative of those found in lung tissue samples; Fe_2_O_3_, Fe_3_O_4_, MnFe_2_O_4_ and CrOOH. As shown in Figure 5, all NP were spherical and presented an average diameter of 25 nm (Fe_2_O_3_, Fe_3_O_4_, MnFe_2_O_4_) or 15 nm (CrOOH) (Figure 5A-D). The NP nature was verified by X-ray diffraction spectra (Figure 5E-H).

### The in vitro effect of the synthesized NP on the macrophage secretome

**Figure 6:**
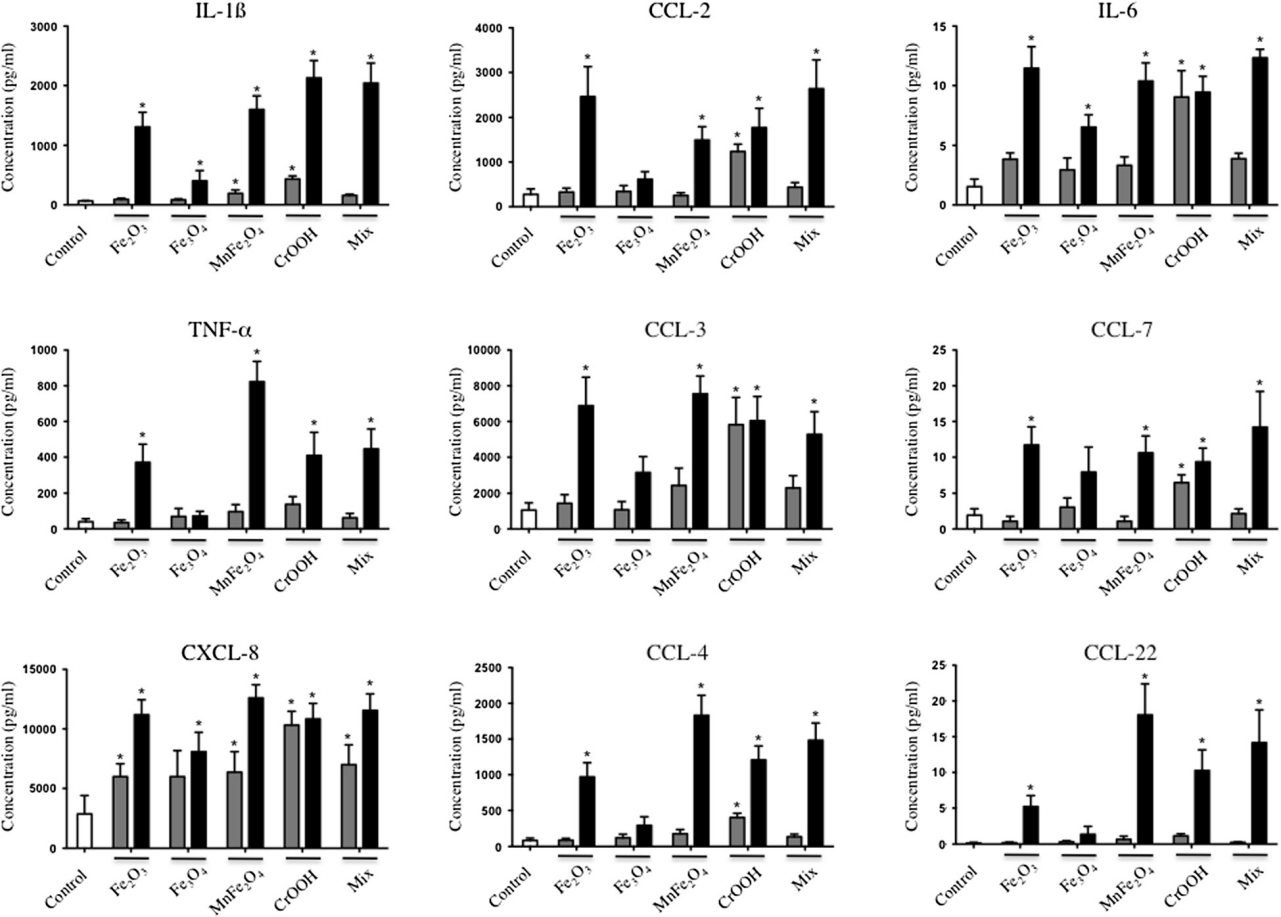
**Pro-inflammatory secretome of THP-1 macrophage exposed for 24 hours to welding-representative NP.** THP-1 were exposed to 5 or 25 μg/cm^2^ NP for 24 hours. Mix is a 1:1:1:1 mixture of the four NP. Macrophage pro-inflammatory secretome was assayed by Luminex. Open bars: Control. Grey bars: 5 μg/cm^2^. Black bars: 25 μg/cm^2^. N = 6 per condition. *: p < 0.05 vs Control condition.

Because NP identified in lungs from welders were in macrophages, we first evaluated the effects of human macrophage exposure to the chemically synthesized representative NP in terms of inflammatory secretome. These experiments revealed the secretion of significantly increased amounts of IL-1ß, TNF-α, CCL-2, −3, −4, and lesser levels of IL-6, CCL-7 and −22 in macrophages exposed to 25 μg/cm^2^ (a concentration representative of occupational exposure [18]) of all but Fe_3_O_4_ NP as compared to unexposed cells (Figure 6). Moreover, CXCL-8 production was also significantly higher in macrophages exposed to all but Fe_3_O_4_ NP at the dose of 5 μg/cm^2^, and to all NP when used at 25 μg/cm^2^ (Figure 6). No modification of the secretion of IL-4, −5, −10, −12, −13, PDGF-AA, PDGF-BB, or TGF-ß was detected, whatever the NP utilized (data not shown). MMP-1 and MMP-7 levels in cell culture supernatants were significantly increased in MnFe_2_O_4_-treated macrophages as compared to unexposed cells (25 μg/cm^2^, Additional file 1: Figure S1). Moreover, exposure to Fe_2_O_3_ NP at 25 μg/cm^2^ induced an increased production of MMP-7 and TIMP-2 as compared to unexposed cells (Additional file 1: Figure S1). Finally, no modification of the expression of MMP-2, −9, −10, TIMP-1, −3, and −4 was observed irrespective of NP exposure (data not shown).

Since welding fumes contain a mixture of NP, we next questioned whether a mix of the most representative NP could have a synergistic effect on the macrophage secretome. These experiments showed that exposure of macrophages to the (1:1:1:1) combination of Fe_2_O_3_, Fe_3_O_4_, MnFe_2_O_4_ and CrOOH NP didn’t produce any synergistic effect in terms of cytokine or MMP/TIMP secretion (Figure 6 and Additional file 1: Figure S1). Finally, as the majority of the welders were smokers, we analyzed if the combined exposure to NP and cigarette smoke could have a synergistic pro-inflammatory effects. These exposures did not demonstrate any synergistic effect of cigarette smoke in macrophages response to NP exposure (Additional file 2: Figure S2 and Additional file 3: Figure S3).

### Effect of macrophage secretome on macrophage migration

Following the identification of the increased number of macrophages in welders lung, the consequence of an autocrine effect of macrophage secretome on the migration capacity of these cells was investigated. Concurrently with our previous data, the secretome obtained from all exposures with the exception of the Fe_3_O_4_ NP-exposed macrophages was able to activate macrophage migration (Additional file 4: Figure S4).

### Expression of pro-inflammatory cytokines in lung tissue sections

**Figure 7:**
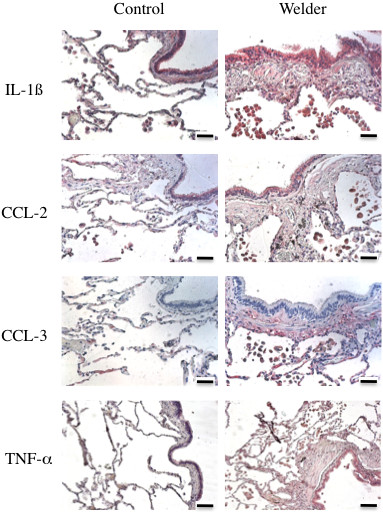
**Expression of IL-1β, CCL-2, CCL-3 and TNF-α in lung tissue sections.** Representative images obtained after immunostaining for IL-1ß, CCL-2, CCL-3 and TNF-α in lung tissue sections from a Control and a Welder. Scale bar: 50 μm. Quantification of the immunostaining is given in Table 4.

To further scrutinize the clinical relevance of the macrophage secretome induction by NP exposure, a series of immuno-histochemistry experiments were performed in lung tissue sections from control and welders, for a subset of cytokines that were highly produced by NP-exposed macrophages. As shown in Figure 7 and quantified in Table 4, the expression of IL-1ß, TNF-α and CCL-3 was significantly higher in lung sections from welders as compared to controls, whereas no difference was observed for CCL-2 expression. For the 3 positive cytokines, and in accordance with NP tissue localization, the staining could be observed in macrophages located in the alveolar lumen as well as in fibrous regions.

**Table 4:** Semi-quantitative score of cytokine expression in lung tissues

	**Controls**	**Welders**	**P value**
	**(n = 7)**	**(n = 10)**	
IL-1β	1.71 ± 1.11	3.60 ± 0.52	0.002
TNF-α	0.83 ± 0.75	3.43 ± 1.81	0.042
CCL2	1.86 ± 1.57	2.00 ± 1.31	ns
CCL3	1.29 ± 1.25	3.00 ± 1.00	0.015

Each value is given as a mean semi-quantitative score^a^ ± SD. ns: not significant; SD: standard deviation.

^a^Semi-quantitative score [0: 0 positive cells/slide; 1: 1–5 positive cells/slide; 2: 5–10 positive cells/slide; 3: 10–50 positive cells/slide; 4: >50 positive cells/slide].

### Effect of macrophage secretome on human lung pulmonary fibroblast activation

Since lung from welders contained an increased number of macrophages not only in alveolar lumen but also in areas of fibrosis, and since NP induced an inflammatory secretome in macrophages *in vitro* that can be similarly detected in situ in macrophages localized in the same areas*,* we next investigated the effects of macrophage secretome on fibroblasts to myofibroblasts differentiation and fibroblasts proliferation. Since, as previously mentioned, the majority of the welders were smokers, these experiments were performed in lung fibroblasts from both non smokers and smokers, to address the specific role of cigarette smoke exposure as well as that of NP. Exposure of lung fibroblasts to the secretome of NP-exposed macrophages revealed neither a modification in their α-smooth muscle actin (α-SMA) staining (Additional file 5: Figure S5A, B and data not shown), nor a change in their proliferation rate over 48 hours (Additional file 5: Figure S5C and data not shown) irrespective of the individual being a smoker or not. Moreover, no modification of the secretion of TGF-ß by fibroblasts exposed to macrophage secretome was observed (data not shown).

## Discussion

In this study a combination of imaging and material science techniques were utilized to comprehensively scrutinize lung tissue samples, and for the first time thoroughly characterize welding-related NP *in situ* in lung tissue from welders. Moreover, given the tissue localization and the effects of such NP on macrophages *in vitro*, the data here strongly suggests that these NP could be responsible at least in part for the pulmonary inflammation observed in welders.

To the best of our knowledge, only a very limited number of studies has been performed in welders, identifying the presence of nanoparticles [19-22]. However, although using the valuable combined (S)TEM/EDX analysis as developed by Abraham and colleagues [23-26], these studies present some major limitations, such as a small number of patient(s) [19,21], or a very specific exposure (aluminum factory, not representative of the general occupational exposure of welders) [20]. Our study in the other hand is the first to provide persuasive evidence not only of the presence of NP *in situ* in human lung tissue samples, but also the identification of their chemical composition. As expected given the occupational exposure of the patients, iron was the major element present in excess in the arc welders evaluated in our study. Indeed, if the exact chemical composition of welding fumes is dependent on the material being welded and the electrode, in manual arc welding fumes and in accordance with our findings, the elemental composition is predominantly iron present as metal oxide NP of the form (M, Fe)_3_O_4_, where M may be substituted for Mn and Cr essentially [9,27-30]. These results led us to chemically synthesize 4 different NP, representative of those found in the welders lungs; 2 speciation of pure iron oxide (Fe_2_O_3_ and Fe_3_O_4_), a mixed Mn/Fe oxide (MnFe_2_O_4_), and an oxyhydroxide CrOOH NP. We chose to generate both Fe_2_O_3_ and Fe_3_O_4_ because, although our data didn’t provide any evidence of the exact speciation of these iron oxides, such species have been largely described in the literature regarding welding fume chemical composition [9,27,30]. Moreover, given the observed co-localization of Mn and Fe signals in our lung samples as well as data from the literature [31], we chose to generate MnFe_2_O_4_ NP as a mix metal oxide relevant to welding fume exposure. Finally, we chose to generate CrOOH oxyhydroid NP that present the same + III oxidation state and particle size in the same range as the other NP. Indeed, FeCr2O4 particles can be synthesized but not at the nanoscale, making them of no interest for our study.

We demonstrated that exposure of macrophages to the synthesized representative NP results in the production of a pro-inflammatory secretome, by itself able to further activate the migration of macrophages *in vitro*. Although we did not conduct experiments to identify the specific protein(s) responsible for the activation of macrophage migration, several candidates present in macrophage pro-inflammatory secretome can be proposed; CCL-2, CCL-3, CXCL-8, as they directly act as efficient monocyte, lymphocyte and neutrophil chemoattractant [32-34]. Moreover, it is important to underline that mediators such as TNF-α and IL-1ß, highly secreted by macrophages in response to NP (Figure 4), could also participate in the perpetuation of inflammation initiated by NP exposure since they have been shown to further induce the expression of chemotactic cytokines such as CCL-2, −3 and CXCL-2, thus sustaining the pulmonary increases of neutrophils, lymphocytes, and macrophages [32,35,36]. Similar events have been described in BAL fluid from animals exposed to welding fumes or other dusts [5,13,37-42]. These events could happen in welders, given that we were able to detect some of these cytokines in lung tissue sections of the patients analyzed in our study. Not all NP produced the same effects *in vitro*, although utilized at the same mass concentration; Fe_3_O_4_ was overall the less reactive NP, particularly as compared to Fe_2_O_3_, which underlines the importance of NP speciation in their individual effects. Such differences in NP effects could be related to different degrees of solubility. However, observation by TEM of 3 months old NP suspensions revealed that all nanoparticles appear similar to those observed in the initial suspension, except for MnFe_2_O_4_ nanoparticles that appear slightly amorphous at their surface (data not shown). This most probably indicates a very small or no solubility at all of the nanoparticle of interest, as we should have observed a diminution of the nanoparticle diameter in case of solubility. This is also in accordance with data from literature demonstrating that iron and chromium oxides are very poorly soluble in water [43-47]. Interestingly, we did not observe any synergistic effect between NP when used as a 1:1:1:1 mix, suggesting a common biological mechanism of action. An LPS contamination of our NP could have been responsible for the M1-like phenotype of THP-1 macrophages *in vitro*[48]. However, the endotoxin content of the NP solutions was evaluated and we didn’t measure any detectable levels of endotoxin, thus ruling out endotoxin contamination (data not shown). Altogether, our data strongly suggests a role for the NP identified in lung tissue samples from welders in the development of their pulmonary inflammation. This could have great implications in other populations of workers occupationally exposed to NP.

As stated previously, the majority of the patients studied here were either current or former smokers which could have been troublesome during the interpretation of the data. Indeed, the presence of NP has been described in cigarette smoke [49]. Therefore, the welding origin of the NP found in welders lungs could be questioned. However, only Al and Si NP were observed in control patients, irrespective of their smoking status, thus ruling out a potential misinterpretation of the findings related to cigarette smoke NP. Moreover, we did not observe any synergistic effect between cigarette smoke and metallic NP exposure *in vitro*. Although we observed only a limited effect of cigarette smoke exposure alone, this underlines the unique effect of welding-related NP.

NP-loaded macrophages were present not only in the alveolar lumen but also in the fibrous tissue of lung tissue sections from welders. In chronic repetitive injury situations leading to fibrosis development (such as welding fume exposure in an occupational context), it is known that inflammation invariably precedes fibrosis [50]. Since we detected a pro-inflammatory secretome in macrophages exposed to NP, we hypothesized that this secretome could induce fibroblast differentiation into myofibroblasts. Indeed, myofibroblasts represent the major cellular effector of fibrogenic reactions, and fibroblasts have long been considered as the only cell type of origin for myofiboblasts [50]. However, the data here did not indicate any myofibroblastic differentiation. These findings could be explained by the absence of TGF-ß production in NP-exposed macrophage supernatant, as TGF-ß is the main cytokine involved in fibroblasts differentiation [50]. Moreover, one can’t rule out the influence of kinetic aspects of our experimental set-up, where macrophages were exposed for 48 hours, leading to a M1-like pro-inflammatory phenotype, without the possibility of developing a later M2 phenotype, where macrophages could act as the primary effectors of later stages of repair and/or later proliferative and remodeling phases. Beside fibroblasts, fibrocytes and epithelial cells have also been described as sources of myofibroblasts [50,51]. Their response to supernatant from NP-exposed macrophages could have been interesting to evaluate given the presence of specific cytokines such as CCL-2 and CCL-3. Indeed, CCL-2 is a chemoattractant for fibrocytes [52], and CCL-3 has been involved in bleomycin-induced recruitment of bone marrow-derived macrophages and fibrocytes, and the subsequent development of pulmonary fibrosis [53]. This should deserve further studies.

## Conclusion

In this study, thanks to the extensive physico-chemical characterization of lung tissue sample from welders, we evidenced the presence of welding-related NP *in situ.* Moreover, our data strongly suggest that these NP could participate in the pulmonary alterations observed in welders. These findings provide the first evidence of a link between human exposure to NP and long-term pulmonary effects.

## Methods

Please refer to online data supplement (Additional file 6) for additional details.

### Patients

Non-tumoral lung tissue sample from 21 arc-welders and matched 21 control patients benefiting surgical resection for primary non-small-cell lung cancer were removed and utilized in the study. All patients were subjected to a thorough occupational questionnaire. All welders had preponderantly arc-welding activities during their whole career, one third of them also performed torch and/or braze welding. The subjects essentially used Tungsten Inert Gas (TIG), Metal Inert Gas (MIG), Metal Active Gas (MAG), and/or Shielded Metal Arc (SMAW), Metal Manual Arc Welding (MMA) techniques. They welded mainly mild steel and/or stainless steel metal pieces, essentially in the following domains of activities; shipbuilding (53%), automotive industry (14%), and sheet metal work (24%). The study was approved by our institutional review board for human studies.

### Histological and immunohistochemical analysis

Paraffin-embedded lung tissue sections (5 μm) were stained with hematoxylin-eosin-saffron (HES) or Perls prussian blue. The localization and severity of fibrosis was evaluated via a semi-quantitative score adapted from Roggli [54]. The presence of other respiratory lesions as well as siderophages and ferruginous bodies were quantified. Mineralogical analysis and quantification of asbestos exposure were performed as previously described [55]. Additionally, immunohistochemical (IHCh) analyses were performed on the deparaffinized tissue sections.

### Characterization of pulmonary NP content

#### Elemental mapping in lung tissue sections

X-ray microfluorescence (μ-XRF) was used to determine the elemental mapping in lung tissue sections. These experiments were performed at the European Synchrotron Radiation Facility (Grenoble, France), on ID21 and ID13 beamlines, at 7.2 and 12.5 keV edges respectively.

#### NP identification in lung tissue homogenates

Lung tissue homogenates were observed using a FEI Tecnai F20 STEM at 200 kV, equipped with an EDX spectrometer.

#### NP identification in lung tissue sections

Paraffin-embedded lung tissue sections were deparaffined and further embedded in Epon resin. Thin sections (300 nm) were stained with Azur II dye and observed via optical microscopy. In addition ultrathin sections (70 nm) counterstained with lead citrate and uranyl acetate were observed using a JEOL1400-TEM at 120 kV, or a FEI Tecnai F20-STEM at 200 kV, equipped with EDX spectrometer for elemental analysis of the identified NP.

### Synthesis of representative NP

Fe_2_O_3_, Fe_3_O_4_, MnFe_2_O_4_ and CrOOH NP were synthesized in aqueous solution using the micro-waved-assisted sol–gel method. Particle nature was determined by X-Ray diffraction (XRD), and their size and shape were evaluated using the Debye-Scherrer equation on several diffraction peaks, together with an analysis of TEM images.

### Exposure of human macrophages to NP

#### Cell culture

Human monocytic THP-1 cells were cultured as previously described [56], and were exposed to 1–25 μg/cm^2^ NP suspensions for 24 hours (non-cytotoxic doses, data not shown). After exposure, the cell supernatant was recovered and centrifuged at 10 000 *g* for 15 minutes to eliminate any NP contamination.

#### Luminex analysis

MILLIPLEX® Human Cytokine/Chemokine, MMP 2 and TIMP 2 kits (Millipore, St-Quentin en Yvelines, France) were used according to manufacturer’s instructions.

#### Macrophage migration

Migration of THP-1 cells in response to supernatant from NP-exposed macrophages was evaluated using a 24-wells Boyden chamber [57].

### Exposure of human fibroblasts to macrophage secretome

Human primary lung fibroblasts were obtained and cultured from a series of control subjects described in [58]. These cells were exposed to NP-exposed macrophages secretome for 48 hours. Fibroblasts expression of α-smooth muscle actin (α-SMA) was then quantified, as well as their proliferation rate (Hoechst assay).

### Statistical analyses

Comparisons between controls and welders were performed using Chi squared, Fisher’s exact or Mann–Whitney nonparametric tests as appropriate (GraphPad Prism software, USA). A p < 0.05 was considered as statistically significant.

## Abbreviations

NP: Nanoparticle; (S)TEM: (Scanning) transmission electron microscopy; EDX: Energy dispersive X-ray; μ-XRF: X-ray microfluorescence; XRD: X-ray diffraction.

## Competing interests

The authors declare that they have no competing interests.

## Authors’ contributions

PA, ASD, JB and SL designed the study. BC performed the face-to-face interview, PA and JCP selected the patients, and PA and FGS performed the histological analysis. MABG performed the mineralogic analyses, and ASD all biological experiments. ASD, BF, JD and SL performed the μXRF experiments, and ASD performed all other imaging experiments. GB and OD synthesized and characterized the NP. PA, ASD and SL drafted the manuscript, and SL and JB were the main additional participants to its further elaboration. All authors read and approved the final manuscript.

## Supplementary Material

Additional file 1: Figure S1 MMP/TIMP levels in supernatant from THP-1 macrophages exposed for 24 hours to welding-representative NP. THP-1 were exposed to 5 or 25 μg/cm² NP for 24 hours. Mix is a 1:1:1:1 mixture of the four NP. Macrophage pro-inflammatory secretome was assayed by Luminex. Open bars: Control. Grey bars: 5 μg/cm². Black bars: 25 μg/cm². N = 6 per condition. *: p < 0.05 vs Control condition. Click here for file

Additional file 2: Figure S2 Pro-inflammatory secretome of THP-1 macrophages exposed for 24 hours to welding-representative NP and cigarette smoke. THP-1 cells were exposed to 25 μg/cm² NP for 24 hours in presence or in absence of 5% cigarette smoke extract (CSE). Mix is a 1:1:1:1 mixture of the four NP. Macrophage pro-inflammatory secretome was assayed by Luminex. Black bars: without CSE. Dashed bars: with CSE. N = 6 per condition. *: p < 0.05 vs respective Control condition. Click here for file

Additional file 3: Figure S3 MMP/TIMP levels in supernatant from THP-1 macrophages exposed for 24 hours to welding-representative NP and cigarette smoke. THP-1 cells were exposed to 25 μg/cm² NP for 24 hours in presence or in absence of 5% cigarette smoke extract (CSE). Mix is a 1:1:1:1 mixture of the four NP. Macrophage pro-inflammatory secretome was assayed by Luminex. Black bars: without CSE. Dashed bars: with CSE. N = 6 per condition. *: p < 0.05 vs respective Control condition. Click here for file

Additional file 4: Figure S4 Quantification of macrophage migration after exposure to NP-exposed macrophage secretome. THP-1 macrophages were exposed to NP-exposed macrophage secretome in Boyden chamber. For each condition, the number of cells that have migrated to the bottom of the membrane was counted in eight fields. Mix is a 1:1:1:1 mixture of the four NP. N = 6 per condition. p < 0.05 vs Control condition. Click here for file

Additional file 5: Figure S5 Characterization of human primary lung fibroblasts response to NP-exposed macrophage secretome. A: Representative images of immunofluorescent staining for α-SMA expression in human primary lung fibroblasts in response to NP-exposed macrophage secretome. Scale bar: 50 μm. TGF-ß was used as a positive control. B: Quantification of α-SMA expression. N = 6 per condition. C: Quantification of human primary lung fibroblasts proliferation in response to NP-exposed macrophage secretome. N = 6 per condition. Click here for file

Additional file 6 Additional Methods section. Click here for file
