# Supplementary figures and images for: Role of metal oxide nanoparticles in histopathological changes observed in the lung of welders
Source: Part Fibre Toxicol. 2014 May 13;11:23. doi: 10.1186/1743-8977-11-23 (PMC4037282; doi:10.1186/1743-8977-11-23)

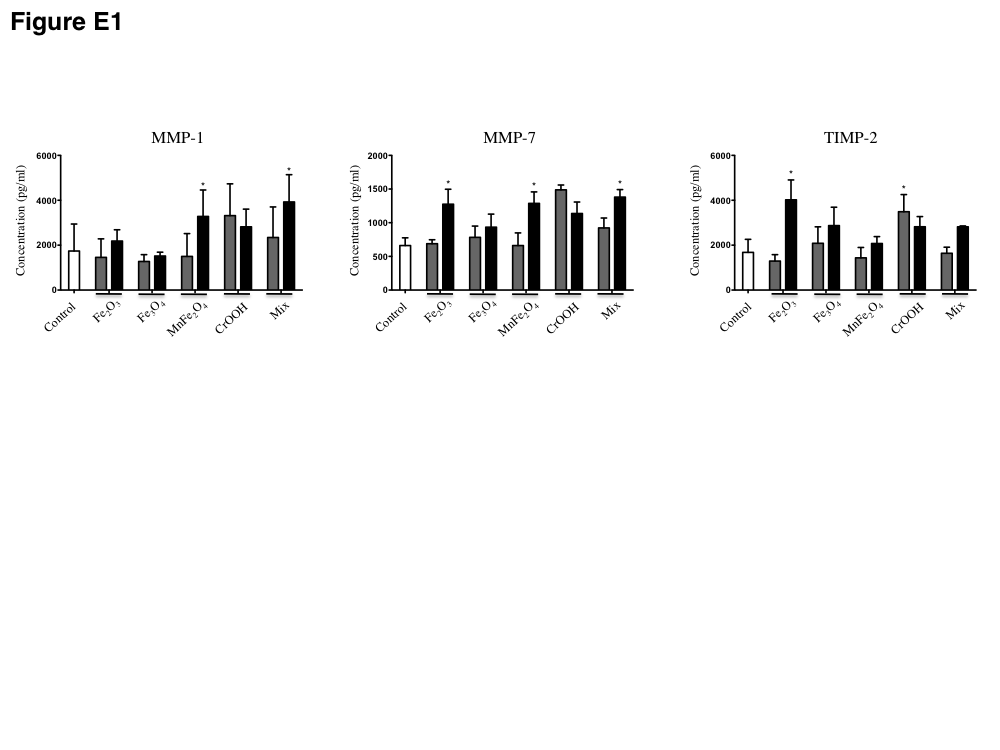

Supplement: Additional file 1: Figure S1 — MMP/TIMP levels in supernatant from THP-1 macrophages exposed for 24 hours to welding-representative NP. THP-1 were exposed to 5 or 25 μg/cm² NP for 24 hours. Mix is a 1:1:1:1 mixture of the four NP. Macrophage pro-inflammatory secretome was assayed by Luminex. Open bars: Control. Grey bars: 5 μg/cm². Black bars: 25 μg/cm². N = 6 per condition. *: p < 0.05 vs Control condition. [file 1743-8977-11-23-S1.tiff]

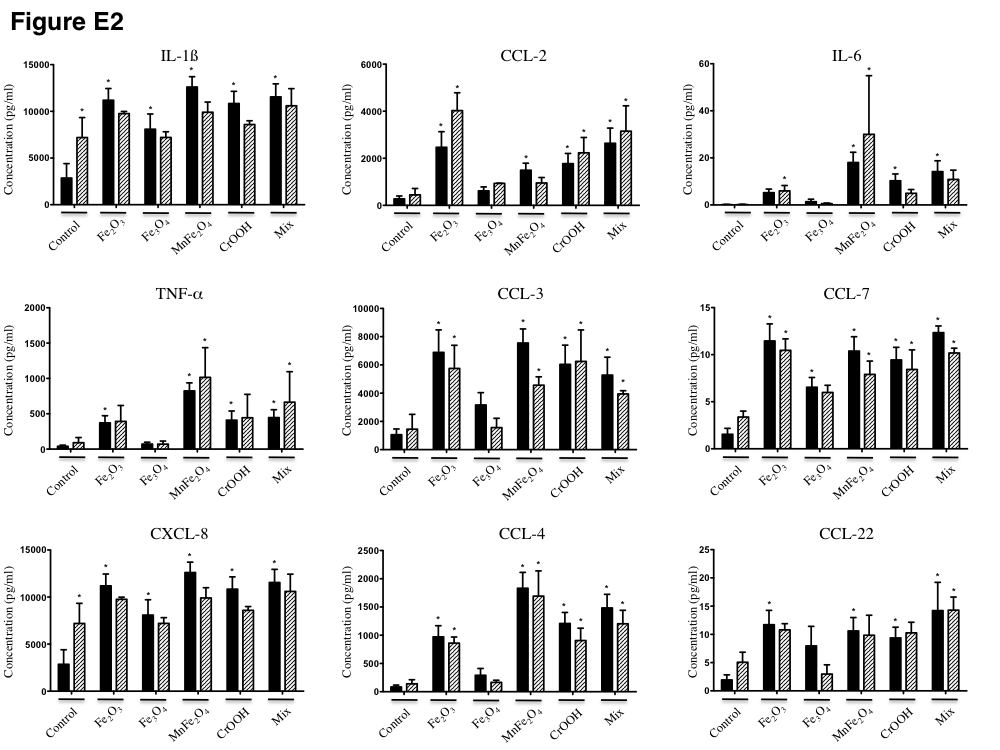

Supplement: Additional file 2: Figure S2 — Pro-inflammatory secretome of THP-1 macrophages exposed for 24 hours to welding-representative NP and cigarette smoke. THP-1 cells were exposed to 25 μg/cm² NP for 24 hours in presence or in absence of 5% cigarette smoke extract (CSE). Mix is a 1:1:1:1 mixture of the four NP. Macrophage pro-inflammatory secretome was assayed by Luminex. Black bars: without CSE. Dashed bars: with CSE. N = 6 per condition. *: p < 0.05 vs respective Control condition. [file 1743-8977-11-23-S2.tiff]

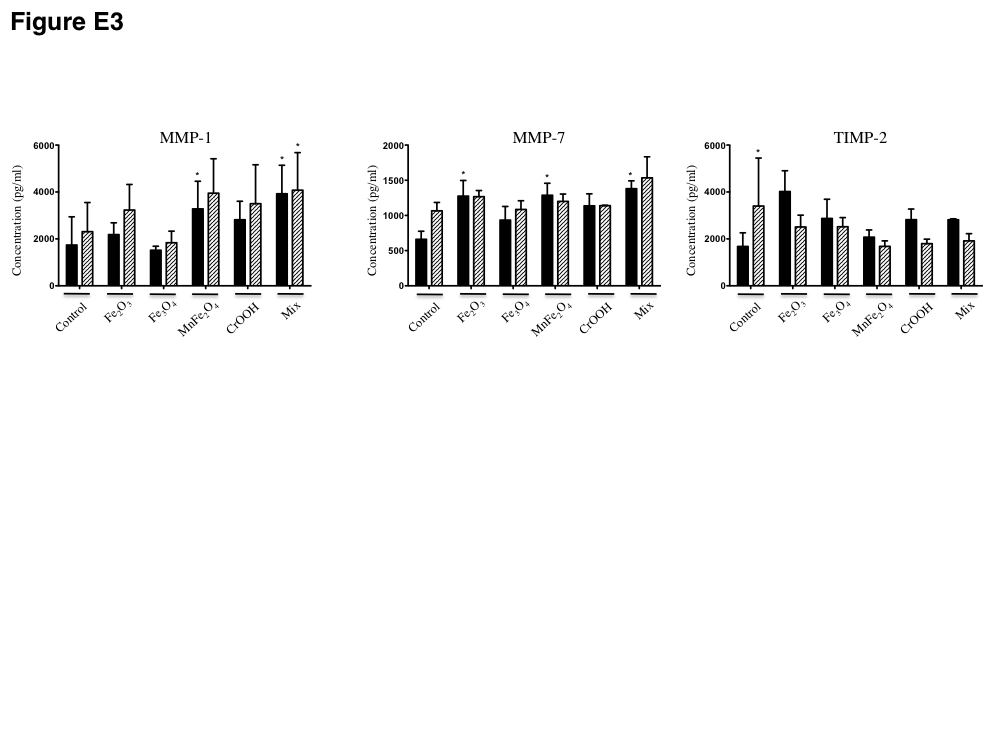

Supplement: Additional file 3: Figure S3 — MMP/TIMP levels in supernatant from THP-1 macrophages exposed for 24 hours to welding-representative NP and cigarette smoke. THP-1 cells were exposed to 25 μg/cm² NP for 24 hours in presence or in absence of 5% cigarette smoke extract (CSE). Mix is a 1:1:1:1 mixture of the four NP. Macrophage pro-inflammatory secretome was assayed by Luminex. Black bars: without CSE. Dashed bars: with CSE. N = 6 per condition. *: p < 0.05 vs respective Control condition. [file 1743-8977-11-23-S3.tiff]

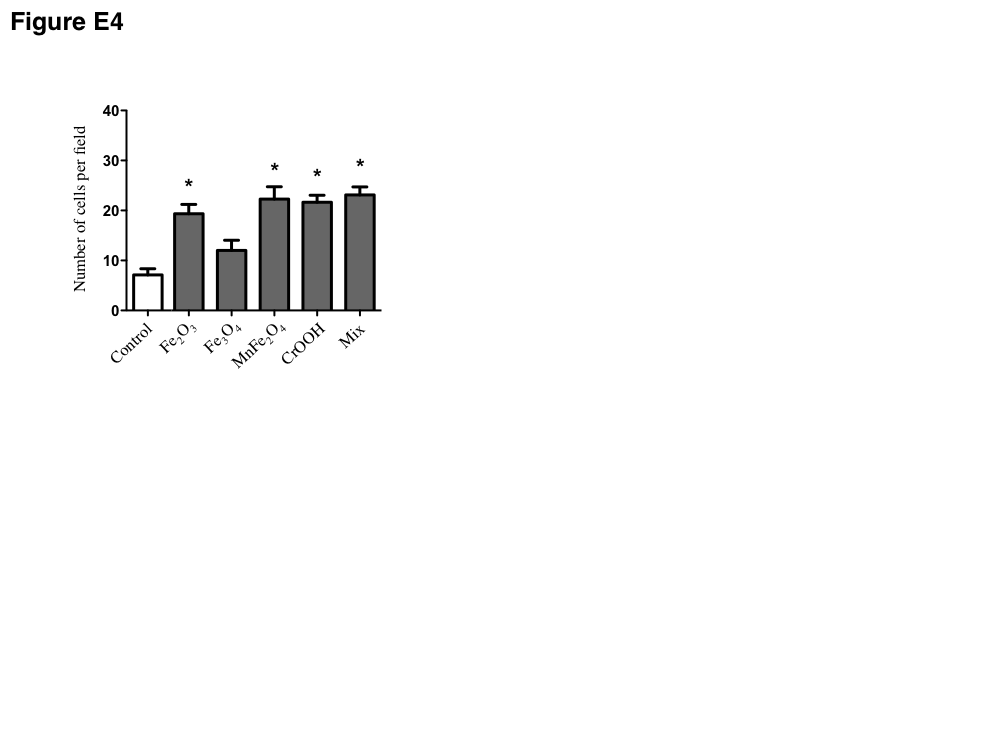

Supplement: Additional file 4: Figure S4 — Quantification of macrophage migration after exposure to NP-exposed macrophage secretome. THP-1 macrophages were exposed to NP-exposed macrophage secretome in Boyden chamber. For each condition, the number of cells that have migrated to the bottom of the membrane was counted in eight fields. Mix is a 1:1:1:1 mixture of the four NP. N = 6 per condition. p < 0.05 vs Control condition. [file 1743-8977-11-23-S4.tiff]

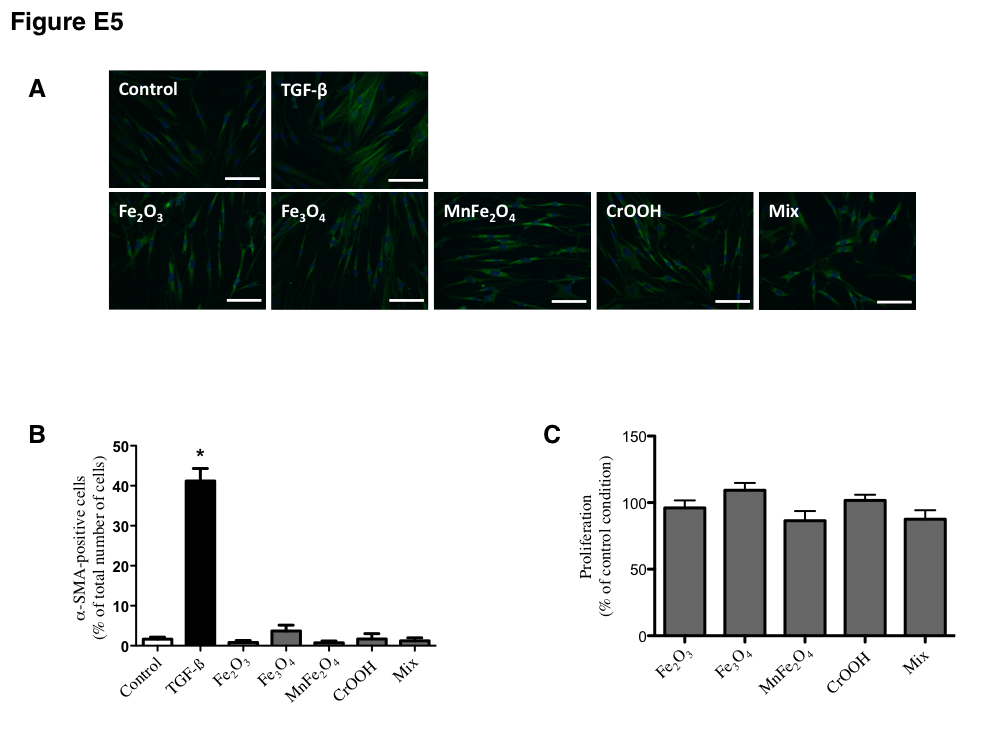

Supplement: Additional file 5: Figure S5 — Characterization of human primary lung fibroblasts response to NP-exposed macrophage secretome. A: Representative images of immunofluorescent staining for α-SMA expression in human primary lung fibroblasts in response to NP-exposed macrophage secretome. Scale bar: 50 μm. TGF-ß was used as a positive control. B: Quantification of α-SMA expression. N = 6 per condition. C: Quantification of human primary lung fibroblasts proliferation in response to NP-exposed macrophage secretome. N = 6 per condition. [file 1743-8977-11-23-S5.tiff]
