# Supplementary material for: Role of metal oxide nanoparticles in histopathological changes observed in the lung of welders
Source: Part Fibre Toxicol. 2014 May 13;11:23. doi: 10.1186/1743-8977-11-23 (PMC4037282; doi:10.1186/1743-8977-11-23)
Supplement: Additional file 6 — Additional Methods section. [file 1743-8977-11-23-S6.docx]

**ONLINE DATA SUPPLEMENT**

**Patients and methods**

Welders and control patients included in the study have been selected from 1994 through 2011, from a series of 358 consecutive patients presenting non-small-cell lung cancer and benefitting from surgical resection in Caen hospital. Welders with less than 10 years of welding activity or exposed to other metallic particles were excluded. For each patient, a non tumoral lung tissue sample was obtained and paraffin-embedded. Information regarding smoking status was obtained during a face-to-face interview to assess smoking classification, i.e never smokers, current smokers and former smokers (quitting smoking at least 1 year before diagnosis). Information on age at onset of smoking, age at smoking cessation, smoking duration and tobacco consumption (cigarettes, cigars and pipes). Finally, a questionnaire including complete job history was completed. On the basis of these information, occupational exposure to particles, especially welding and painting activities, metallic dust, crystalline silica and asbestos exposure, was determined by consensus between two occupational hygienists not informed about mineral particles and fibers analysis in normal lung tissue. Asbestos exposure was ascertained if the questionnaire concluded on definite occupational exposure to asbestos for more than 10 years, and/or when the asbestos bodies (AB) count was higher than 1000 per gram of dry lung tissue, a value indicative of non-trivial (usually occupational) asbestos exposure [1, 2]. Unexposed subjects were those with no occupational or environmental exposure identified from assessment with the questionnaire and with an AB count less than 1000 per gram of dry lung tissue.

**Immunohistochemistry**

Deparaffinized tissue sections were labeled with specific antibodies directed against human CD68 (M0814, DAKO, Trappes, France), IL-1β (ab2105, Abcam, UK), CCL-2 (HPA01963, Sigma, France), CCL-3 (SC-1381, Santa Cruz, Germany), TNF-α (SC-52746, Santa Cruz, Germany), or α-SMA (ab46805, Abcam, UK).

**Metallic elements characterization,** **localization and quantification by X-ray Micro-Fluorescence (µXRF)**

The microprobe size was 0.3x0.3 µm^2^ at ID21 at 7.2 keV (allowing elements lighter than Fe to be detected), and 0.3x0.3 µm^2^ at ID13 at 13.0 keV (allowing elements lighter than Zn to be detected). The lung tissue section was scanned to acquire a two-dimensional point-by-point image. In order to minimize the contribution of any unwanted elastic scattering the energy-dispersive high-purity Germanium detector was mounted in the horizontal plane perpendicular to the beam. The measurements were carried out in vacuum (10^-5^ mbar) in order to minimize the absorption of air. The signal obtained for each element of interest was reported to that of sulphur for semi-quantification between samples [3, 4].

**Nanoparticle synthesis, characterization and preparation**

*Magnetite nanoparticles synthesis Fe_3_O_4_*

A first solution was prepared by pouring 2.78 g of iron (II) chloride (14 mmol) in 5.3 mL of distillated water and then adding 2.41 mL of a 2.0 mol.L^-1^ HCl solution. Then 1.46 mL of a 4.1 mol.L^-1^ iron (III) chloride solution (6 mmol) were added under constant stirring and a nitrogen bubbling. The temperature of the solution was the set to 70°C with an oil bath and its pH was progressively set to 12 by dropwise addition of a 25 wt.% aqueous solution of tetramethylammonium hydroxide. A green-grey precipitate first appeared. The pH = 12 value was maintained during 20 minutes and the precipitate turned black. The solution was then transferred in Teflon liners and aged at 200°C for 1h in a microwave oven (Milestone).

*Maghemite nanoparticles synthesis γ-Fe_2_O_3_*

The magnetite nanoparticles Fe_3_O_4_ prepared above were oxidized into maghemite nanoparticles γ-Fe_2_O_3_ according to the following procedure. Before drying the magnetite nanoparticles were isolated from the supernatant solution with a magnet and redispersed in 12 mL of a 2 mol.L-1 nitric acid solution for 30 minutes. The supernatant solution is again removed and the particles are transferred into 12 mL of a boiling solution containing 2.42 g of Fe(NO_3_)_3_,9H_2_O (6 mmol) for an additional hour under stirring.

*Jacobsite nanoparticles synthesis MnFe_2_O_4_*

A degassed solution of hydrochloric acid with a concentration of 0.5 mol.L^-1^ was prepared and heated in an oil bath at 70°C. Then, were successively added, 1.5 g of Mn(NO_3_)_2_,4H_2_O (6 mmol) and 4.8 g of Fe(NO_3_)_3_,9H_2_O (12 mmol). The pH of the solution was then set to 11 under argon bubbling by the dropwise addition of a 2 mol.L^-1^ sodium hydroxide solution and maintained at this value for 10 minutes. The obtained nanoparticles were finally transferred in an autoclave and aged 2 hours at 200°C in a conventional oven.

*Grimaldiite nanoparticles synthesis CrOOH*

A degassed solution of hydrochloric acid with a concentration of 0.5 mol.L^-1^ was prepared and heated in an oil bath at 70°C. Then 4.8 g of Cr(NO_3_)_3_,9H_2_O (12 mmol) were added. The pH of the solution was then set to 3.5 under argon bubbling by the dropwise addition of a 2 mol.L^-1^ sodium hydroxide solution and maintained at this value for 10 minutes. The obtained nanoparticles were finally aged 3 hours at 200°C in a microwave oven.

*X-ray diffraction (XRD) characterization*

XRD measurements were performed with a Brücker D8 X-ray diffractometer operating in the Bragg Brentano reflection mode equipped with a Nickel filter to select the Cu Kα radiation. The data were collected in the 10 – 70° 2θ range with 0.05° steps. Pattern analyses were performed using EVA software (Brucker AXS) and the ICDD DD View PDF-4+ 2009 RDB base. Nanoparticle sizes D_hkl_ have been calculated using the Debye-Scherrer formula: D_hkl_ = (0.9λ)/(b_hkl_cosθ) with λ = (2λ_Kα1_+ λ_Kα2_)/3 and b_hkl_ is the FWHM for the (hkl) diffraction line corrected from the instrumental broadening (b_hkl_² = FWHM²-b_inst_² with b_inst_=0.03°). In the Debye-Sherrer equation the value K = 0.9 was chosen for it is the commonly used value for an unknown particles morphology and for the use of the peak width at half height measurement.

*Preparation of nanoparticle suspension*

Nanoparticles were washed several times prior to use. Chemical composition, size and shape were ascertained by TEM-EDX. NPs suspensions (2 mg/mL) were prepared in ultrapure water, sonicated four times for 20 sec at 65 W with an external ultrasonic probe (Ultrasonic Processor, Fisher Bioblock Scientific, Illkirch, France) and stored at -80°C. Just before use, aliquots were sonicated 10 min in ultrason bath (Elmasonic S30H).

**Human monocytic THP-1 cells culture**

Human monocytic THP-1 cells (ATCC TIB-202) were cultured in RPMI medium (Life technologies, Villebon sur Yvette, France) supplemented with 10% heat inactivated fetal bovine serum, 50 U/mL penicillin, 50 μg/mL streptomycin, 0.05 mM 2-mercaptoethanol. Cells were maintained at 37°C in a humidified 5% CO_2_/95% air incubator. One passage before exposure, THP-1 cells were cultured in DMEM medium and seeded into well plates at a concentration of 2.5x10^5^ cells/cm^2^ in DMEM medium with 30 ng/mL of PMA (Sigma-Aldrich, St-Quentin Fallavier, France) to induce macrophage differentiation [5]. After 24 hours, cells were washed three times with DMEM medium without serum and exposed to NP suspensions, from 1 to 25 µg/cm² during 24 hours. These concentrations were assessed as non cytotoxic (data not shown).

For cigarette smoke exposure experiments, briefly, mainstream cigarette smoke from one 2R4F research cigarette (Kentucky Tobacco Research and Development Center, Kentucky) was bubbled in 50 ml of culture medium to a obtain cigarette smoke extract (CSE). This CSE was further diluted at 5% in culture medium to expose macrophages. New CSE was freshly prepared for each day of experiment.

**Migration assays**

Migration assays were performed using a 24-well Boyden chamber (BD Biosciences, Le Pont de Claix, France) as previously described [6]. The top and bottom chambers were separated by a polycarbonate filter with 3 µm pores. The top wells were loaded with 200 µl of THP-1 cells at a concentration of 1.10^6^ cells/ml in DMEM with 10% SVF and 30 ng/mL of PMA. After 24 hours, cells were rinsed twice with PBS, and 200 µL of DMEM without SVF was added. The bottom wells were loaded with 700 µL of supernatant from NP-exposed macrophages. Plates were incubated at 37°C for 24 hours. The filter was rinsed with PBS, the top side was scraped twice, and the bottom one was fixed and stain with Diff-Quick (Grifols France, Meyreuil, France). Migrated cells were counted in eight fields from images captured using an optical microscope with a x20 objective lens.

**REFERENCES**

1. Meirelles GS, Kavakama JI, Jasinowodolinski D, Nery LE, Terra-Filho M, Rodrigues RT, Neder JA, Napolis LM, Bagatin E, D'Ippolito G, Muller NL: **Pleural plaques in asbestos-exposed workers: reproducibility of a new high-resolution CT visual semiquantitative measurement method.** *J Thorac Imaging* 2006, **21:**8-13.

2. Case BW, Abraham JL, Meeker G, Pooley FD, Pinkerton KE: **Applying definitions of "asbestos" to environmental and "low-dose" exposure levels and health effects, particularly malignant mesothelioma.** *J Toxicol Environ Health B Crit Rev* 2011, **14:**3-39.

3. Merigoux C, Briki F, Sarrot-Reynauld F, Salome M, Fayard B, Susini J, Doucet J: **Evidence for various calcium sites in human hair shaft revealed by sub-micrometer X-ray fluorescence.** *Biochim Biophys Acta* 2003, **1619:**53-58.

4. Bussy C, Cambedouzou J, Lanone S, Leccia E, Heresanu V, Pinault M, Mayne-L'hermite M, Brun N, Mory C, Cotte M, et al: **Carbon Nanotubes in Macrophages: Imaging and Chemical Analysis by X-ray Fluorescence Microscopy.** *Nano Lett* 2008, **8:**2659-2663.

5. Lanone S, Rogerieux F, Geys J, Dupont A, Maillot-Maréchal E, Boczkowski J, Lacroix G, Hoet PH: **Comparative toxicity of 24 manufactured nanoparticles in human alveolar epithelial and macrophage cell lines.** *Particle and fibre toxicology* 2009, **6:**14.

6. Wagsater D, Zhu C, Bjorck HM, Eriksson P: **Effects of PDGF-C and PDGF-D on monocyte migration and MMP-2 and MMP-9 expression.** *Atherosclerosis* 2009, **202:**415-423.

**FIGURE LEGENDS**

**Figure E1: MMP/TIMP levels in supernatant from THP-1 macrophages exposed for 24 hours to welding-representative NP.**

THP-1 were exposed to 5 or 25 µg/cm² NP for 24 hours. Mix is a 1:1:1:1 mixture of the four NP. Macrophage pro-inflammatory secretome was assayed by Luminex. Open bars: Control. Grey bars: 5 µg/cm². Black bars: 25 µg/cm². N=6 per condition. *: p<0.05 vs Control condition.

**Figure E2: Pro-inflammatory secretome of THP-1 macrophages exposed for 24 hours to welding-representative NP and cigarette smoke.**

THP-1 cells were exposed to 25 µg/cm² NP for 24 hours in presence or in absence of 5% cigarette smoke extract (CSE). Mix is a 1:1:1:1 mixture of the four NP. Macrophage pro-inflammatory secretome was assayed by Luminex. Black bars: without CSE. Dashed bars: with CSE. N=6 per condition. *: p<0.05 vs respective Control condition.

**Figure E3: MMP/TIMP levels in supernatant from THP-1 macrophages exposed for 24 hours to welding-representative NP and cigarette smoke.**

THP-1 cells were exposed to 25 µg/cm² NP for 24 hours in presence or in absence of 5% cigarette smoke extract (CSE). Mix is a 1:1:1:1 mixture of the four NP. Macrophage pro-inflammatory secretome was assayed by Luminex. Black bars: without CSE. Dashed bars: with CSE. N=6 per condition. *: p<0.05 vs respective Control condition.

**Figure E4: Quantification of macrophage migration after exposure to NP-exposed macrophage secretome.**

THP-1 macrophages were exposed to NP-exposed macrophage secretome in Boyden chamber. For each condition, the number of cells that have migrated to the bottom of the membrane was counted in eight fields. Mix is a 1:1:1:1 mixture of the four NP. N=6 per condition. p<0.05 vs Control condition.

**Figure E5: Characterization of human primary lung fibroblasts response to NP-exposed macrophage secretome.**

**A:** Representative images of immunofluorescent staining for α-SMA expression in human primary lung fibroblasts in response to NP-exposed macrophage secretome. Scale bar: 50µm. TGF-ß was used as a positive control. **B:** Quantification of α-SMA expression. N=6 per condition. **C:** Quantification of human primary lung fibroblasts proliferation in response to NP-exposed macrophage secretome. N=6 per condition.
